# Sonic Hedgehog Signaling Pathway in Endothelial Progenitor Cell Biology for Vascular Medicine

**DOI:** 10.3390/ijms19103040

**Published:** 2018-10-05

**Authors:** Amankeldi A. Salybekov, Ainur K. Salybekova, Roberto Pola, Takayuki Asahara

**Affiliations:** 1 Department of Regenerative Medicine Science, Tokai University School of Medicine, 143 Shimokasuya, Isehara, Kanagawa 2591193, Japan; amansaab0@gmail.com (A.A.S.); ainurasaab@gmail.com (A.K.S.); 2 Department of Medicine, Fondazione Policlinico Universitario A. Gemelli IRCCS, Università Cattolica del Sacro Cuore, 00168 Rome, Italy; roberto.pola@unicatt.it

**Keywords:** sonic hedgehog, endothelial cells, endothelial progenitor cells, canonical signaling, non-canonical signals, extracellular vesicles

## Abstract

The Hedgehog (HH) signaling pathway plays an important role in embryonic and postnatal vascular development and in maintaining the homeostasis of organs. Under physiological conditions, Sonic Hedgehog (SHH), a secreted protein belonging to the HH family, regulates endothelial cell growth, promotes cell migration and stimulates the formation of new blood vessels. The present review highlights recent advances made in the field of SHH signaling in endothelial progenitor cells (EPCs). The canonical and non-canonical SHH signaling pathways in EPCs and endothelial cells (ECs) related to homeostasis, SHH signal transmission by extracellular vesicles (EVs) or exosomes containing single-strand non-coding miRNAs and impaired SHH signaling in cardiovascular diseases are discussed. As a promising therapeutic tool, the possibility of using the SHH signaling pathway for the activation of EPCs in patients suffering from cardiovascular diseases is further explored.

## 1. Introduction

Endothelial progenitor cells (EPCs) were first isolated from adult peripheral blood (PB) in 1997 [1]. Circulating EPCs are bone marrow (BM)-derived cells that incorporate into the foci of physiological or pathological neovascularization to form new blood vessels [2]. In situ, circulating EPCs localize at sites of neovascularization and differentiate into endothelial cells (ECs). This process is similar to vasculogenesis, which involves the formation of new blood vessels during embryonic development. In adult organisms, a reservoir of EPCs from bone marrow contributes to postnatal neovascularization [3]. The discovery of EPCs has expanded the field of vascular biology beyond the conventional EC biology and now is applied for studies involving organ regeneration and vascular diseases. Several research investigations related to regenerative medicine have aimed at elucidating the differentiation cascade of EPCs and their role in the origin of vascular development. Two types of EPCs have been described, namely, hematopoietic including circulating EPCs and non-hematopoietic EPCs. Few non-hematopoietic EPCs include endothelial out-growth cells (EOCs) and tissue resident c-kit+/CD45− progenitor cells [2,4,5]. Several signaling cascades are common to hematopoietic EPCs, non-hematopoietic EPCs and differentiated ECs. However, distinct signaling mechanisms associated with the differentiation and subsequent bioactivity of EPCs reveals their unique role in vascular development and pathology during the postnatal period.

The Hedgehog (HH) signaling pathway coordinates embryonic and postnatal angiogenesis as well as organogenesis. In vertebrates, during the early embryonic development, HH morphogens play a crucial role in the development of organs such as limbs [6,7], neurons (axon elongation and astrocyte development) [8,9,10] and cardiac and vascular structures for septal cardiogenesis, angiogenesis and vasculogenesis [11,12,13,14,15]. Mammals have three genes with homology to the Hh gene, namely, Sonic hedgehog (SHH), Indian hedgehog (IHH) and Desert hedgehog (DHH). The SHH signaling pathway has drawn research interest as it induces postnatal vasculogenesis under homeostasis and pathological conditions [16,17,18,19,20].

The present review focuses on the recent research developments in EPC biology, namely, (i) canonical and non-canonical SHH signaling pathways in EPCs and EC, related to homeostasis, (ii) SHH signal transmission by extracellular vesicles such as exosomes containing single-strand non-coding miRNAs and (iii) impaired SHH signaling in cardiovascular diseases. In conclusion, the role of activated SHH signaling pathway in EPCs as a promising therapeutic tool for cardiovascular patients has been explored.

### 1.1. SHH Signaling Pathways in Vascular Development

**Figure 1:**
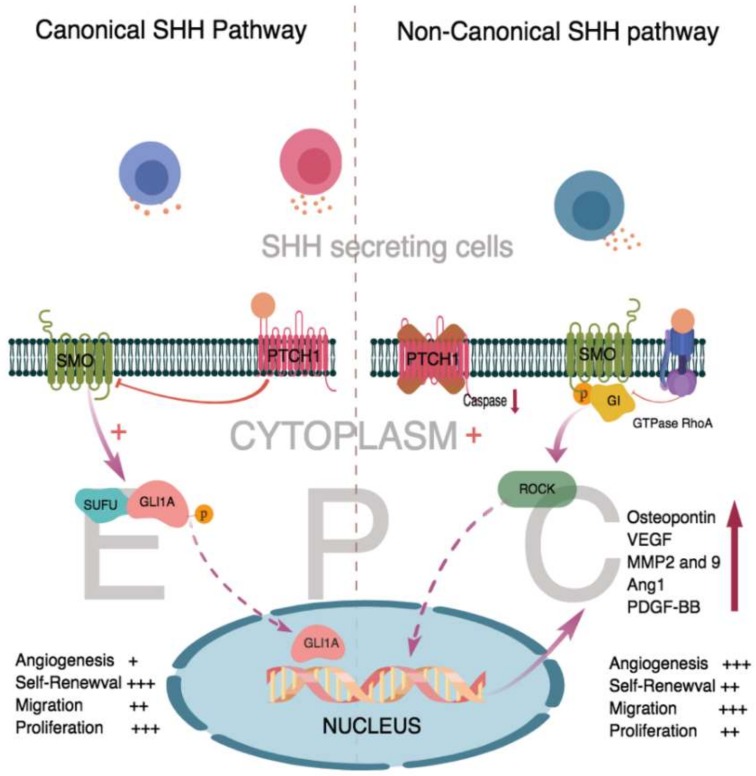
Canonical and Non-Canonical Sonic hedgehog (SHH) signaling pathway in endothelial progenitor cells (EPCs) and endothelial cells (ECs). SHH molecule binds and activate the 12-pass transmembrane cell surface receptor Patched 1 (PTCH 1) that inhibits the smoothened (SMO) receptor to activate the binding of suppressor of fused (SUFU) and glioma-associated oncogene homolog1-A (GlLI 1A) to form a complex by autophosphorylation. EPCs are mostly activated by the non-canonical SHH signaling pathway in angiogenesis.

Classical canonical SHH signaling is initiated by the binding of SHH morphogens to 12-pass transmembrane cell surface receptor Patched 1 (PTCH 1) present on the target cell [21]. De-repressed smoothened (SMO) protein, a central signal transducer of the SHH signaling pathway, activates the downstream glioma-associated oncogene homolog (GLI) family of transcription factors, namely, GLI1, GLI2 and GLI3 (Figure 1). Direct binding of suppressor of fused (SUFU) to GLI transcription factors negatively regulates GLI-mediated transcription by inhibiting their nuclear entry and ability to transcribe DNA [22]. At the nuclear level, accumulation of GLI transcription factors activate target genes associated with proliferation (Cyclin-D1, MYC), apoptosis (BCL-2), angiogenesis (ANG)1/2, PDGF-BB, VEGF)), epithelial-to-mesenchymal transition (SNAIL) and stem cell self-renewal (NANOG, SOX2) [21,23,24].

Recent research in the field of vascular biology reveals significant contribution of the non-canonical SHH signaling rather than canonical SHH signaling in vascular development under homeostasis or pathological conditions [13,17,25]. In endothelial cells, SHH morphogens trigger the non-canonical SHH signaling pathway leading to vascular differentiation, maturation and function [14,17,18,19,26,27]. Stimulation of a confluent endothelial monolayer of cells with recombinant SHH molecules but not IHH or DHH morphogens, resulted in a change in the overall morphology of cells from the typical EC “cobblestone” shape to a swirling pattern of elongated cells oriented in bundles, a pattern characteristic of activated ECs engaged in angiogenesis [25,28].

In one of the non-canonical SHH signaling pathways, SHH proteins could activate membrane RhoA GTPase in a SMO/GI protein-dependent manner [25]. The membrane RhoA family of small GTPases play an important role in cell migration and invasion by orchestrating guanine nucleotide exchange factors and GTPase-activating protein complexes [29]. Cell migration mediated by RhoA GTPase signaling is specific in different cell lines. More than 20 members of the Rho family have been discovered and are divided into classical and non-typical types. The RhoA GTPase responsible for the activation of the Gi/SMO complex in ECs is less studied [30]. In vitro, the tube formation assay showed that Hh proteins stimulated tubulogenesis by inducing rapid activation of RhoA levels in human umbilical-vein ECs via SMO and GI proteins, while inhibition of RhoA activation hindered tube formation [25,31]. Binding of SHH molecule to RhoA GTPase brought forth a 3-fold increase in the concentration of RhoA GTPase, thereby delivering a strong angiogenic signal required to activate the non-canonical SMO-dependent SHH signaling pathway for the maturation of EPCs to ECs (Figure 1) [25,27,31].

Survival of ECs is mediated by a decrease in caspase-3 activity that inhibits the PTCH1 pro-apoptotic function in a SMO-independent manner [25,27]. A combined transcriptomic and proteomic approach identified a 101-gene endothelial signature that could be used to characterize endothelial commitment. The HH-interacting protein regulates GLI-dependent canonical HH signaling and is a strong negative regulator of the late endothelial progenitor cells (LEPCs). Knockdown of hedgehog-interacting protein in LEPCs improves the angiogenic activity and enhances their survival under oxidative stress [32]. Thus, exogenous administration of SHH could be beneficial for the survival of EPCs after the onset of ischemic events.

SHH morphogens activate the Rho-associated protein kinase (Rho/ROCK) pathway and enhances the expression of downstream matrix metalloproteinase 9 (MMP-9), osteopontin (OPN) and platelet derived growth factor (PDGF-BB) (Figure 1), which are essential for SHH-induced angiogenesis in vitro [14,25,27]. An in vivo mouse corneal angiogenesis model was used to investigate the potential involvement of SMO, the Rho/ROCK pathway, MMP-9, OPN and the GLI transcription factors in SHH-induced angiogenesis. Pellets containing phosphate-buffered saline (PBS) or cyclopamine (SMO protein inhibitor) alone and in combination with SHH, were implanted and the extent of angiogenesis was evaluated by in vivo fluorescein-BS-1 lectin perfusion. PBS+ SHH significantly increased angiogenesis, which was inhibited by cyclopamine. To analyze the role of the Rho/ROCK pathway in SHH signaling, pellets containing PBS, SHH, Y27632 (ROCK inhibitor), or SHH+Y27632 (ROCK inhibitor) were implanted. Significantly enhanced downstream targets of ROCK, MMP-9 and OPN dependent angiogenesis were observed in the SHH treated group compared to those in the SHH+Y27632 treated group that showed no significant changes [27]. A mouse corneal angiogenesis model demonstrated SHH-dependent PDGF-BB–induced pericyte cell recruitment, essential for the maturation of newly formed blood vessels [14].

Taken together, the above results prove the vital role of SHH morphogens in activating the non-canonical rather than the canonical signaling pathway in EPCs and ECs, thereby regulating migration, angiogeneic bioactivity, survival and maturation.

Gupta et al., recently reported a non-canonical SHH signaling pathway with an indirect effect of SHH molecules in mediating angiogenesis in EPCs and ECs. They demonstrated that in vitro, the effect of SHH on the proliferation and migration of ECs was limited by direct incubation of SHH in culture but was significantly enhanced in the presence of conditioned media from SHH-treated fibroblasts or stromal cells. In addition, treatment of fibroblasts with SHH significantly enhanced the expression profile of angiogenic growth factors including PDGF-B, VEGF-A, hepatocyte growth factor (HGF) and insulin-like growth factor (IGF). Among these, PDGF-B was most predominantly upregulated and might have contributed to the formation of large neo-vessels associated with SHH-induced indirect angiogenesis [26]. In vivo, in a corneal angiogenesis model, administration of exogenous SHH showed no significant difference in corneal angiogenesis between endothelial-specific smoothened knockout (eSmoNull) and eSmoWT mice. An in vivo hind-limb ischemia (HLI) model in eSmoNull and eSmoWT mice was used to assess the importance of SMO-dependent SHH signaling in SHH-mediated angiogenesis in ECs. The study demonstrated equal recovery in both eSmoNull and eSmoWT mice in terms of perfusion ratio, limb motor function, limb necrosis and blood vessel formation [16,26]. The results suggest the role of fibroblast-derived pro-angiogenic genes in indirectly activating angiogenesis that is independent of SMO proteins in EPCs and ECs.

### 1.2. SHH Signal Transmission by Extracellular Vesicles

Extracellular vesicles (EVs) are lipid bilayered structures, 30–150 nm in size, enclosing cargo containing messenger ribonucleic acid (mRNA), microRNAs (miRNAs), growth factors and proteins for transfer into recipient cells [33,34]. From biogenesis to release, all the EVs share three main stages, namely, (i) outward budding and fission that occur at the plasma membrane, (ii) formation of early endosome, packaging and sorting in endoplasmic reticulum to form exosomes or EVs and (iii) release of exosomes or EVs into the extracellular space [33,35]. In mammals, SHH is secreted on two distinct types of EVs/exosomes exhibiting distinct protein and RNA composition to directly or indirectly activate downstream target genes [36]. Vyas et al. [36] isolated two distinct exosome fractions, P150 and P450, from full-length SHH transfected HEK293T cells using differential ultracentrifugation technique. The EV pools were derived from an endocytic origin as the expression of the endocytosis protein RAB was found to be higher in both the fractions.

**Figure 2:**
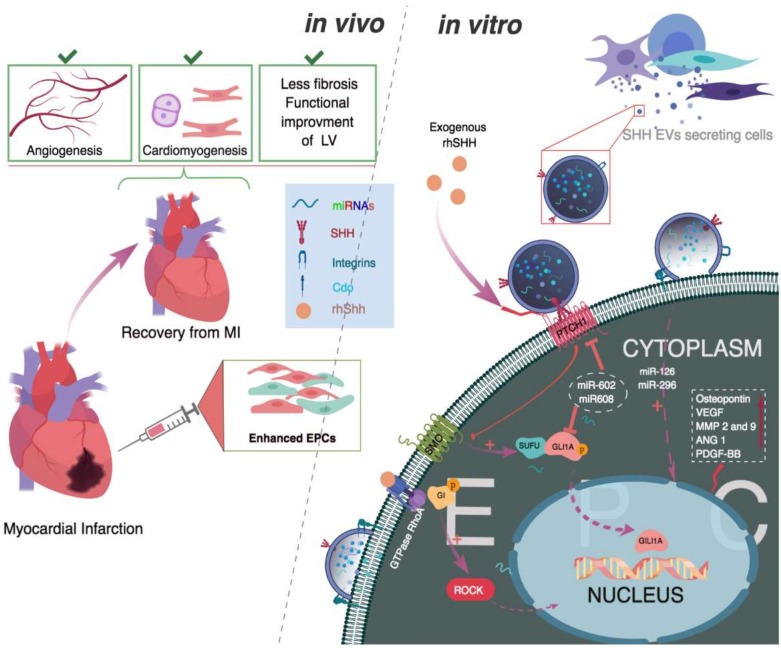
Sonic Hedgehog (SHH) molecule or SHH secreted on extracellular vesicles (EVs) mediated functional improvement of endothelial progenitor cells (EPCs). In vivo, administration of exogenous SHH molecules increased vasculogenic EPCs. Transplantation of enhanced EPCs was beneficial for recovery in myocardial infarction (MI).

Regulation of the SHH signaling pathway by EV-derived miRNA is divided into three levels of activation, namely, binding of miRNA with membrane surface receptors or proteins (PTCH1 or activates Rho GTPase), subsequent binding with cytoplasmic proteins GLI1, GLI 2 and GLI 3 and the nuclear level of activation, which is the strongest among the three levels [37,38,39] (Figure 2). Chondrocytes isolated from osteoarthritis patients showed enhanced expression of SHH, PTCH1, GLI 1 and metalloproteinase-13, which positively correlated with the overexpression of miRNA-602 and miRNA-608 responsible for the activation of the above mentioned three levels [40]. EVs derived from cancerous cells exhibited significantly enhanced expression of SHH and GLI and positively correlated with the microvascular density (MVD) of tumor tissue, suggesting the important role played by SHH morphogens in cancer cell growth and metastasis by promoting the formation of microvascular network [41].

According to in vitro studies, SHH secreted on EVs activated downstream signal transduction in EPCs by the canonical PTCH1-GLI1 signaling or through the non-canonical signaling mediated by pro-angiogenic miRNAs, integrin-linked kinases and ROCK dependent pathway [27,31,42,43] (Figure 2). In an in vivo study, SHH-coding vector transfected CD34+ cells or EPC exosomes had strong vasculogenic potential and aided the recovery of myocardial infarcted tissues by enhancing angiogenesis and reducing left ventricular fibrosis [17]. Analysis of transcriptional profile revealed overexpression of miRNA-126a and miRNA-296 in EPC-derived exosomes and an in vivo study showed improved self-renewal and vasculogenic functions in EPCs that enhanced angiogenesis in a murine HLI model (Table 1) [44]. Taken together, the above results indicate the importance of EVs derived SHH molecules or miRNAs in postnatal angiogenesis and tumor metastasis. Current research focuses on elucidating the functions of SHH-EVs derived miRNAs to develop novel therapeutic drugs for the benefit of patients suffering from cardiovascular ischemic diseases (Table 1).

### 1.3. Impaired SHH Signaling in Cardiovascular Diseases

Cardiovascular diseases (CVD) contribute to almost 32% of all deaths worldwide. Among them, ischemic diseases are a leading cause of morbidity and mortality [50,51]. EPCs in the peripheral bloodstream of patients with morbidities such as atherosclerosis, diabetes mellitus (DM), hypertension and obesity, together with risk-associated factors (smoking and western diet) are impaired in number, quality and function [21,52,53]. Preclinical studies related to DM, acute myocardial infarction (AMI), wound healing and chronic vascular inflammatory diseases indicated increased activation of endogenous SHH signaling pathway in non-treated group compared to that of the treated group, wherein exogenous administration of SHH promoted functional recovery of EPCs, resulting in enhanced angiogenesis, cardiomyogenesis and wound healing [26,54,55]. Additional studies have demonstrated the contribution of SHH molecules in the process of neovascularization in ischemic tissues in animal models of HLI and AMI, where the biological effects were brought about by an EPC enriched cell population, CD34+ cells [17,19,56] (Table 2). In a study, human CD34+ cells isolated from healthy volunteers or patients suffering from Burger’s disease were treated with SHH after administration of granulocyte colony-stimulating factor (G-CSF). The results indicated enhanced expression of pro-angiogenic genes in a dose-dependent manner by SHH protein, particularly in patient-derived CD34+ cells compared to that of CD34+ cells derived from healthy controls, in the presence or absence of G-CSF [17]. In vivo, streptozotocin-induced DM type 1 mice exhibited impaired tube-forming ability, migration and mobilization of EPCs compared to that of the healthy control group. EPCs of DM type 1 mice showed cross-talk between SHH and phosphatidylinositol-4,5-bisphosphate 3-kinase (PI3K)/AKT pathways, which decreased the activity of AKT and increased GSK-3β activity, resulting in the degradation of the SHH pathway transcription factor GLI1/GLI2 [57]. In human pancreatic cancer stem cells, the PI3K/AKT and SHH signaling pathways cooperate to inhibit the transcription factor GLI1/GLI2 to decrease cell viability and to induce apoptosis [58]. Compared to the control littermates, type 1 diabetic mice with myocardial infarction showed impaired SHH pathway with significantly decreased SHH, PTCH 1 and GLI1 protein levels in the myocardial tissue, resulting in extended left ventricle infarct size and reduced capillary density leading to cardiac dysfunction [59]. GLI1 protein is essential for regulating cell-cycle, survival, apoptosis, angiogenesis and migration of cells [24]. Patients with cardiovascular diseases, levels of endogenous SHH-PTCH1-GLI1 protein complex is decreased in EPCs because of chronic inflammation and risk factors. In such patients, administration of exogenous SHH molecules might aid in the recovery of PTCH1-GLI1 complex to beneficially improve the EPC functional profile [57,59,60]. Studies related to the transplantation of EPCs in ischemic diseases such as AMI, ischemic cardiomyopathy, heart failure, peripheral arterial disease (PAD) and stroke have documented lack of recovery of EPCs from ischemia in aged and DM patients compared to that of the control group. These results indicate a decrease in the concentration of PTCH1-GLI1 molecule or sensitivity of SHH signaling pathway receptors to EPCs to be dependent on the severity, age, type and timing of the disease [61,62,63]. Accordingly, studies related to aged mice showed decreased expression of SMO in the skeletal muscles [64].

### 1.4. Therapeutic Application of SHH Signaling in Cardiovascular Diseases

In patients with end-stage ischemic disease, the effectiveness of interventional reperfusion therapy is hampered [67,68,69]. In such patients, EPC therapy is a promising therapeutic option for improving angiogenesis, vasculogenesis and contemporary organ preservation. Transplantation G-CSF mobilized EPCs are safe and feasible for patients with advanced coronary artery disease or PAD, who are not amenable to surgical or percutaneous revascularization [70,71,72]. However, efficacy of EPC mobilization with G-CSF is very low in DM and previously EPC mobilized patients because of the impaired quality and quantity of EPCs [73]. In cardiovascular patients who previously underwent cell mobilization, SHH-mediated activation of EPC is a promising option for the functional recovery of EPCs [17]. Results of preclinical studies related to the therapeutic implementation of SHH proteins or activation of the SHH signaling pathway beneficially improved in several ischemic disease models such as AMI [15,43], myocardial ischemic—reperfusion, HLI [17,23,45,74], stroke [75], diabetic wound healing [44], skeletal myogenesis [76], osteogenesis and bone tissue formation [77] Table 2. The advantages and disadvantages of the therapeutic application of SHH molecules in cardiovascular related diseases are reviewed below.

### 1.5. Ischemic Heart Diseases

A young human heart is composed of cardiomyocytes (approximately 18%), ECs (24%) and mesenchymal cells or fibroblasts 58% [78]. Recent research with sophisticated cardiac single cell preparation and immunohistochemistry analysis revealed that the endothelial cells constitute over ~51–54%, hematopoietic-derived cells ~3% and fibroblasts under equating to ~11% of the total cells of the heart when assuming ~30–33% of the cells to be cardiomyocytes [79]. As endothelial cells are the major cellular component of the cardiac tissue, together with SHH molecules, they could be therapeutically exploited to benefit patients suffering from cardiovascular diseases (Figure 2). In the therapeutic application of EPCs +SHH molecules, SHH signaling preserve cardiac function and improve cardiac recovery in the context of myocardial ischemia. To this end, combination therapy using intramyocardial SHH gene transfer and AMD3100-induced progenitor-cell mobilization significantly improved the recovery of cardiac function after the onset of MI in mouse. Mice administered with the combination therapy demonstrated increased MVD, reduced left ventricle fibrosis area and significantly enhanced expression of SDF-1α after MI [80]. One of the limitations associated with the broad use of SHH molecules in therapy is its short half-life in the body. In this regard, controlled delivery of SHH morphogens to the ischemic myocardium using a coacervate delivery system prolongs the therapeutic efficacy of SHH molecules. The coacervate delivery system protects the SHH molecules from degradation and promotes controlled release of the molecule for a period of over 3 weeks [81]. Transplantation of EPCs together with controlled delivery of SHH morphogens to the ischemic myocardium would be a promising therapeutic tool to treat cardiovascular diseases. Various therapeutic applications of endogenous and exogenous activation of SHH signaling in AMI and myocardial ischemia-reperfusion–induced injury models are summarized in Table 2.

### 1.6. Peripheral Arterial Diseases

Majority of the therapeutic effects of SHH signaling were investigated using PAD model in mouse and rat. The importance of the therapeutic application of SHH is based on its regulatory function associated with the development of limbs during embryogenesis and postnatal skeletal myogenesis, angiogenesis, vasculogenesis and neurogenesis [6,7,40]. Endogenous SHH promotes infiltration of macrophages. Myocytes of mice deficient for SHH signaling showed enhanced expression of VEGF-A with a transient increase in angiogenesis compared to that of the healthy controls. These results indicate the ability of SHH molecules to limit inflammation and angiogenesis indirectly by signaling in myocytes, whereas exogenous administration of SHH molecules promoted ischemia-induced angiogenesis and skeletal myogenesis (Table 2) [19,74,76]. In a HLI model in aged mice, combination of SHH gene transfer and transplantation of bone marrow-derived EPCs (BM-EPCs) could effectively promote angiogenesis and muscle regeneration compared to monotherapy with BM-EPCs. Treatment with SHH+ EPCs enhanced the incorporation of EPCs into the host blood vessels, suggesting a promising role of SHH in increasing the migration of transplanted EPCs into the site of ischemia to enhance angiogenesis and vasculogenesis. In vivo, combined treatment with SHH+ EPCs significantly reduced apoptosis in EPCs and increased proliferation of myoblast proliferation after the onset of HLI Table 2 [55]. These results suggest that combined treatment with SHH and EPC could accelerate proliferation of quiescent myogenic stem cells (satellite cells) and promote myotube formation by accelerating their fusion, eventually leading to the formation of mature myofibers supplied with new blood vessels.

### 1.7. Post DM Complications

Patients with DM are at increased risk of cardiovascular diseases and associated clinical complications. DM is estimated to affect 552 million people worldwide by 2030 [82]. A recent cohort study involving 1.9 million people with type 2 diabetes and incidence of cardiovascular diseases showed a strong positive association between type 2 diabetes and PADs, ischemic stroke, stable angina, heart failure and non-fatal MI [83]. Thus, post diabetic clinical complications are a global burden that requires new therapeutic strategies. Animal models with DM type 1 along with AMI or HLI were used for preclinical investigation of Hh signaling. Results from these experiments showed downstream HH signaling proteins such as PTCH1, SMO, GLI 1, GLI 2 and GLI 3 to be functionally impaired, indicating impairment of SHH signaling pathway in cardiovascular diseases. Combined treatment with exogenous recombinant SHH molecule and EPCs significantly improved the histological and functional parameters in experimental animals that indicated enhanced expression of PTCH1, SMO and GLI 1, GLI 2 and GLI 3 as detected by the transcriptome analysis [57,59]. In a diabetic animal model, administration of nanoscale polymer SHH conjugates accelerated wound closure via activation of SHH pathway. The beneficial effects of SHH treatment were evident based on wound revascularization detected using immunohistochemistry that quantified endothelial cells, CD31 and formation of neovascular structures in the wound tissues [54].

Following post-stroke, immediate treatment with SHH pathway agonists significantly increased the expression of vasculogenesis-related factors including VEGF, FGF and ANG, together with SHH signaling proteins PTCH1, GLI 1, GLI 2 and SMO. SHH treatment improved the neurological scores, reduced the infarct volume, promoted angiogenesis and neuronal survival in the ischemic boundary zone [75]. Strikingly, delayed treatment (post 3-8 days) with SHH pathway agonists enhanced the recovery of locomotor behavioral and cognitive function within 1 month of the onset of stroke, suggesting that a prolonged treatment window for potential treatment strategy can be modulated for SHH pathway post-stroke Table 2 [66].

## 2. Conclusions

SHH signaling pathway is a key regulator of postnatal vasculogenesis. In cardiovascular patients, endogenous SHH signaling pathway is impaired to varying degrees depending on the severity of the disease such as AMI, stroke and PAD due to the mitigated angiogenic potential of EPCs. Most of the preclinical experimental studies related to CVDs have shown that treatment with SHH molecules and EPCs could significantly enhance angiogenesis, vasculogenesis, cardiomyogenesis, skeletal myogenesis and neurogenesis. These therapeutic effects were evident even with delayed treatment. Cross-talk between SHH- and other signaling pathways, or key regulators such as wingless/integrated (WNT) or Notch may also be involved in maintaining the functional activity of EPC in angiogenesis. Thus, further research is needed to elucidate the cross-talk of SHH signaling with WNT, Notch, or protein kinase A (PKA). To date, most of the data available related to SHH signaling are derived from in vitro experiments and are difficult to be verified in vivo because of the presence of numerous signaling pathways and the associated cross-talk between them in an experimental organism. Thus, further research is required to explore the possibility of exploiting SHH signaling as a therapeutic tool for treating CVDs.

## Figures and Tables

**Table 1:** Summary of pro-angiogenic and anti-senescence micro-RNAs (miRNAs) in endothelial progenitor cells (EPCs) and endothelial cells (ECs).

Name of the miRNA	Expression	Target Cells	Outcome	Target Genes	Ref.
miR-126-3p	Up	EPC and EC	In vitro, presence of miR126-3p increases the length of tube formation. In vivo, it increases the MVD in animal models with HLI.	VEGF, ANG-1, ANG-2 and MMP-9	[44]
miR-106b-25	Up	EC, EPC, SCA-1 and BMMSC	Increased tube formation capacity. Overexpression of individual members of the miR-106b-25 cluster increases viability, proliferation and migration of ECs.	VEGF, SCA-1 and FLK-1	[45]
miR-126	Down	EPC, SCA-1 and Lin-	Silencing miR-126 in animal models with HLI increases mobilization of EPC, SCA-1 and Lin- cells from bone marrow to the site of injury and enhanced angiogenesis.	SDF-1	[46]
miR-10A and miR-21	Down	EPC	miR-10A and miR-21 regulates senescence in EPCs by suppressing the expression of HMGA 2.	HMGA 2	[47]
miR-361	Down	EPC	In vitro, KO of miR-361-5p restores VEGF levels and angiogenic activities in diseased EPCs. In vivo, it promotes blood flow and recovery of ischemic limbs in mice.	VEGF	[48]
miR-34a	Down	EPC	Overexpression of miR-34a significantly enhanced senescence and impairment in EPC that paralleled with 40% reduction in SIRT1. KO of SIRT1 by siRNA decreased angiogenesis and increased senescence in EPCs.	SIRT1 and FOXO1	[49]

Abbreviations: EPC—endothelial progenitor cell; EC—endothelial cell; HLI—hind-limb ischemia; BMMSC—bone marrow mesenchymal stromal cells; MVD—microvascular density; Lin—lineage negative cells; KO—knock-out; VEGF—vascular endothelial growth factor; Ang—angiopoietin; MMP—matrix metalloproteinase; SCA—stem cells antigen; FLK—fetal liver kinase; SDF—stromal cell-derived factor; HMGA—high mobility group AT-hook; SIRT—sirtuin (silent mating type information regulation 2 homolog); FOX—Forkhead box.

**Table 2:** Endogenous and Exogenous Activation of Sonic Hedgehog (SHH) Signaling in Cardiovascular Diseases.

Disease Model	SHH Pathway and Cell Tx.	Results	Ref.
AMI	Activation of endogenous and exogenous SHH signaling by SHH–modified human CD34+ cells and its exosomes	Treatment with SHH–modified human CD34+ cells reduced infarct size, increased border zone capillary density and improved cardiac function; EF, FS, compared with unmodified CD34 cells or cells transfected with the empty vector.	[15]
AMI and Chronic MI	Exogenous recombinant SHH administration and gene transfer of naked DNA encoding human SHH	MI fibrosis size and apoptotic cardiomyocytes were reduced. MVD was increased. SHH gene transfer enhanced the contribution of bone marrow–derived endothelial progenitor cells to myocardial neovascularization.	[56]
Myocardial Ischemia-Reperfusion–Induced Injury	Activation of endogenous HH signaling and administration of exogenous recombinant SHH	Reduced apoptosis, fibrosis and increased vascularization. Exogenous SHH administration reduced apoptosis, increased vascularization and reduced	[60]
Post-myocardial ischemic-reperfusion injury	Activation of endogenous HH signaling and exogenous recombinant SHH administration	Exogenous SHH administration significantly increased vasculogenesis-related factors including VEGF, FGF and ANG as well as the SHH signal proteins including PTCH-1, GLI 1, GLI 2 and SMO.	[65]
HLI	SHH-treated human G-CSF mobilized EPCs locally injected into the HLI muscles	Incubation of CD34+ cells with exogenous SHH molecule significantly increased vasculogenesis-related factors including VEGFA, VEGFB, HGF and Pecam 1 as well as the SHH signal proteins including PTCH-1, GLI 1, GLI 2 and SMO at dose 1μg/mL. In vivo significant increase in angiogenesis and vasculogenesis and recovery by blood perfusion following HLI.	[17]
HLI	SHH conditioned fibroblast media or exosomes	PDGF-B, VEGF-A, HGF and IGF. PDGF-B was significantly upregulated and contributed to MVD. Improved blood flow perfusion after HLI.	[23]
HLI	Combined treatment with SHH and EPC	Increased incorporation of EPC within host vessels, reduced apoptotic of EPC and initiated the generation of new myocytes.	[55]
Diabetic wound healing	Administration of exogenous nanoscale polymer encapsulated SHH	Accelerated diabetic-induced wound closure.	[44]
DM type 1 mouse was inducted AMI	SHH + EPCs Tx	EPC migration, tube forming ability and mobilization were impaired in diabetic mice compared to that of control. In vivo administration of the SHH pathway receptor agonist improved all the above. SHH molecules significantly increased capillary density and blood perfusion in the ischemic hind-limbs of diabetic mice.	[57]
Ischemic Stroke	Administration of exogenous SHH	SHH treatment results in enhanced functional recovery both in locomotor function and in cognitive function 1-month post-stroke. Increased the cerebral blood flow map by arterial spin labeling and immunohistochemistry á-smooth muscle actin and CD31 immunostaining.	[66]

Abbreviation: Tx—transplantation; AMI—acute myocardial infarction; EF-ejection fraction; FS—fractional shortening; SV—stroke volume; MVD—microvascular density; HLI—hind-limb ischemia; DM—diabetes mellitus; FGF—fibroblast growth factor; IGF—insulin growth factor; VEGF—vascular endothelial growth factor; HGF—hepatocyte growth factor; PDGF-B—platelet derived growth factor-B; ANG—angiopoietins.
